# Past, Present, and Future Vulnerability to Dengue in Jamaica: A Spatial Analysis of Monthly Variations

**DOI:** 10.3390/ijerph17093156

**Published:** 2020-05-01

**Authors:** Sheika Henry, Francisco de Assis Mendonça

**Affiliations:** Department of Geography, Federal University of Parana, Curitiba 81531-980, Brazil; chico@ufpr.br

**Keywords:** dengue, climate change, Jamaica, vulnerability assessment, WADI framework

## Abstract

Over the years, Jamaica has experienced sporadic cases of dengue fever. Even though the island is vulnerable to dengue, there is paucity in the spatio-temporal analysis of the disease using Geographic Information Systems (GIS) and remote sensing tools. Further, access to time series dengue data at the community level is a major challenge on the island. This study therefore applies the Water-Associated Disease Index (WADI) framework to analyze vulnerability to dengue in Jamaica based on past, current and future climate change conditions using three scenarios: (1) WorldClim rainfall and temperature dataset from 1970 to 2000; (2) Climate Hazard Group InfraRed Precipitation with Station data (CHIRPS) rainfall and land surface temperature (LST) as proxy for air temperature from the Moderate Resolution Imaging Spectroradiometer (MODIS) for the period 2002 to 2016, and (3) maximum temperature and rainfall under the Representative Concentration Pathway (RCP) 8.5 climate change scenario for 2030. downscaled at 25 km based on the Regional Climate Model, RegCM4.3.5. Although vulnerability to dengue varies spatially and temporally, a higher vulnerability was depicted in urban areas in comparison to rural areas. The results also demonstrate the possibility for expansion in the geographical range of dengue in higher altitudes under climate change conditions based on scenario 3. This study provides an insight into the use of data with different temporal and spatial resolution in the analysis of dengue vulnerability.

## 1. Introduction

Dengue fever is a vector-borne disease that is transmitted by the bite of an infected mosquito. The principal vector responsible for the transmission of the disease is the *Aedes aegypti* mosquito, which is mostly found in urban areas. To a lesser extent, the *Aedes albopictus*, found in rural areas, is also a dengue vector [1]. The virus is comprised of four different serotypes (DENV 1, DENV 2, DENV 3, and DENV 4). Infection from a particular serotype results in immunity to that serotype [2]. Symptoms of dengue include fever and at least two of the following: headache, joint pain, muscle or bone pain, rash, nose bleeding, or pain behind the eyes. Dengue can also manifest in dengue haemorrhagic fever or dengue shock syndrome [3]. 

The dengue vector is mostly found in the tropics between latitudes 35° N and 35° S, but the mosquito has also been detected between 45° N and 40° S in summer [4]. The Centre for Disease Control and Prevention (CDC) estimates that over 40% of the population worldwide reside in areas that are at risk of dengue transmission [5]. In fact, the clinical manifestation of dengue was estimated at 96 million cases annually, with yearly infections amounting to about 390 million [6]. Even though dengue is an economic burden in many countries due to loss of productivity [7] and deaths [8], it is referred to as one of the “neglected tropical diseases” (NTD) by the WHO [9]. As per the Dengue Prevention and Control Strategy 2012–2020 report, the lack of political will, global coordination efforts and research, are some of the factors that result in the disease being classified as NTD [9].

Furthermore, dengue propagation continues due to the failed elimination strategies that were implemented in the 20th century to rid the planet of the *Aedes aegypti* mosquito [10]. The eradication program coordinated by PAHO in 1947 to eliminate the dengue vector was estimated at USD 1,681,775,000 and was only successful in about twenty countries in 1962 [11]. By 1963, there was a re-emergence of the vector in areas where eradication occurred. 

Jamaica is one of the countries with sporadic cases of dengue fever. The island has recorded about seven major outbreaks between 1995 and 2018. Approximately 986 suspected and confirmed cases, along with 13 deaths, were recorded in 2019 [12]. In 2016, about 2297 cases of dengue and two deaths were reported [12]. However, in 2012, approximately 4670 cases were reported, while 2827 cases were recorded in 2010. As it relates to 1995 and 1998, roughly 1884 and 1551 cases were reported, respectively [13]. With climate change, the rate of dengue transmission is likely to increase. So far, experts have theorized that roughly 3.8% of deaths from dengue worldwide are linked to climate change [14].

In Jamaica, some studies on dengue fever have been conducted. As it relates to knowledge, attitudes, and practices of dengue [15,16,17], the lack of preventative measures by respondents to reduce transmission was highlighted. Also, research shows the circulation of all four dengue serotypes on the island [18]. Analysis of the serotypes regarding the antigenic structure of the virus [19] illustrates how antibodies have neutralized the agent. In another study [20], domestic containers were identified as one of the breeding sites for the *Aedes agegypti* mosquito in some regions in the country (Portland, St. Ann, and St. Catherine). A study on vulnerability to dengue was conducted [21], but this was based on a perception-based approach in Montego Bay. The results showed that dengue cases were located close to water bodies and informal settlements with improper water storage containers.

A regional dengue analysis involving other Caribbean islands demonstrated a seasonal trend in the occurrence of dengue, especially during El Nino and El Nino +1 years in Jamaica [22]. Furthermore, the Economic Commission for Latin America and the Caribbean (ECLAC) evaluated the economic impact of climate change on health in Jamaica between 2011 and 2050 on dengue, leptospirosis, and gastroenteritis [23], and dengue was the only disease that was predicted to increase.

As demonstrated above, studies on dengue in Jamaica have been conducted; however, there is paucity in the use of Geographic Information Systems (GIS) and remote sensing technology to analyze the disease. This research will attempt to fill this gap. As a result, the objective is to conduct a spatio-temporal vulnerability assessment of dengue fever in Jamaica using a modified approach of the Water-Associated Disease Index (WADI) that was used in a study in Malaysia [24] based on past, current, and future climatic conditions using three scenarios. Scenario 1 is based on the WorldClim rainfall and temperature dataset for 1970 to 2000 (A) while Scenario 2 uses Climate Hazards Group Infrared Precipitation with Stations (CHIRPS) and Land Surface Temperature (LST) (B) as a proxy for air temperature from the Moderate Resolution Imaging Spectro-radiometer (MODIS) for the period 2002 to 2016. The third scenario includes maximum temperature and rainfall using the RCP 8.5 climate change scenario for 2030 (C). The following hypotheses will be used to guide this research: (1) urban areas will have higher vulnerability to dengue than rural areas, and (2) the geographical range for transmission of dengue will likely change under climate change conditions. The goal of this research is to show the usefulness of publicly available spatial data in the study of dengue in countries like Jamaica, where access to time series data of the disease is sparse.

The WADI approach incorporates socio-economic and environmental conditions as susceptibility and exposure indicators, respectively. The conceptual framework is based on an ecological aspect to study the spread of “water-associated pathogen” [24] that affects human health. Within this regard, secondary data available within the public domain that can be used to represent socio-economic and susceptibility conditions are utilized in order to determine regions with high and low vulnerability to water-associated diseases such as dengue. This is especially relevant in countries where dengue data might not be accessible to researchers.

## 2. Materials and Methods

### 2.1. Study Area

**Figure 1:**
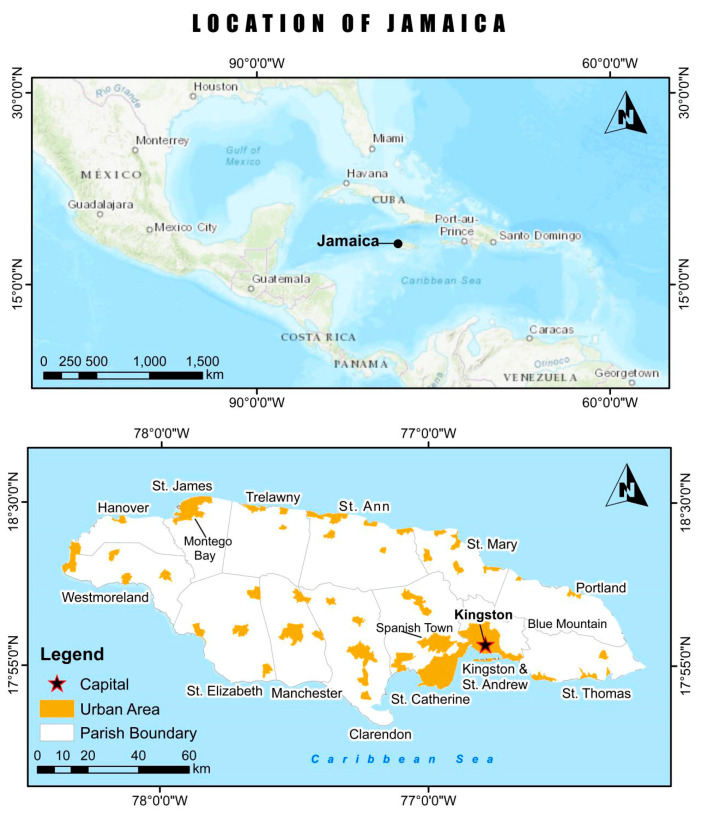
Location of Jamaica [27]. Urban area based on the 2011 Enumeration Districts from the Statistical Institute of Jamaica (STATIN) [26].

Jamaica is an island located in the Caribbean Sea (Figure 1), which is about 145 km south of Cuba and 190 km west of Haiti [25]. It has an area of about 10,991 km^2^ and is situated between 18°15′ N latitude and 77°20′ W longitude. The country is divided into fourteen parishes, with Kingston as its capital. According to the most recent census collected by the Statistical Institute of Jamaica (STATIN), approximately 2,697,983 people reside on the island [26].

Based on the Koppen–Geiger classification, Jamaica has an equatorial climate (humid tropical) along with the following climate subtypes: Af (fully humid—tropical rainforest), Am (tropical monsoon) and Aw (desert—tropical savannah) [28]. Average annual rainfall in 2015 was estimated at 1307 mm while the average annual temperature was 28.4 °C for the same year.

### 2.2. Data Used for the Analysis

The data used in this study (Table 1, Table 2 and Table 3) were obtained within the public domain as access to time series dengue data at the community level in Jamaica proved futile during data collection for a PhD thesis. Socio-economic data were obtained from the 2011 Census collected by the Statistical Institute of Jamaica [26] and was used in all the scenarios. Some of the freely available data were available in tabular format, which was converted into GIS format for analysis.

In the first scenario, monthly cumulative precipitation and maximum temperature data were derived from WorldClim Version 2 between 1970 and 2000 at 1 × 1 km [29]. This dataset is based on the interpolation of mean monthly in situ data. The second scenario included maximum rainfall from CHIRPS [30] and mean LST from MODIS MOD11A2 [31] from 2002 to 2016. MOD11A2 used to generate LST has a resolution of 1 × 1 km, which is a substitute for air temperature. The LST data were provided by NASA but accessed via Google Earth Engine (GEE). The CHIRPS data, on the other hand, were obtained from Climate Engine and has a resolution of 0.05° based on a monthly time scale.

The maximum temperature and rainfall data for 2030 based on the RCP 8.5 climate change scenario downscaled at 25 km based on RegCM4.3.5 Model were obtained from the Caribbean Community Climate Change Centre (CCCCC). CCCCC is responsible for climate change and adaptation in the Caribbean. The maximum temperature for 2030 was converted from Kelvin to degrees Celsius in Excel. Also, the total precipitation rate for 2030 was converted to mm per month [27].

The 1998 land cover data with a scale of 1:100,000 were obtained from the Forestry Department of Jamaica [32]. This was updated in ArcGIS to represent the 2016 land use with the Editor tool based on Google imagery.

The dataset with health centres was obtained from the Ministry of Health of Jamaica in GIS format.

### 2.3. Methodology

The WADI framework that was utilized in Malaysia to assess vulnerability to dengue was applied in this research [24]. This framework produces a vulnerability index as a function of exposure, susceptibility and adaptive capacity. Within this regard, susceptibility incorporates the socio-economic factors that make the population at risk of being infected with the dengue virus. Exposure on the other hand, relates to environmental conditions that lead to the propagation of the disease. The adaptive capacity refers to the “ability of a system to adjust to climate change to moderate potential damages, to take advantage of opportunities, or to cope with the consequences” [33]. Consequently, this study will assess vulnerability to dengue virus infection. Table 1 shows the exposure indicator utilized.

The susceptibility indicator incorporated components such as age, housing quality (squatter settlement), access to piped water, sanitation, garbage collection, and lack of education (Table 2).

Additionally, access to health care and the percentage (%) of females completing secondary school education per parish were only utilized for the adaptive capacity as there was no data on public health intervention (Table 3).

#### Index Construction

The data was manipulated in ArcGIS while the spatial multi-criteria evaluation (SMCE) was completed in ILWIS (the Integrated Land and Water Information System). ILWIS allows for the manipulation of spatial data and was developed by the International Institute for Aerospace Survey and Earth Sciences in The Netherlands.

The exposure, susceptibility and adaptive capacity indicators were used to create new layers with values between 0 and 1 based on a modified approach used in the Malaysian study [24] and the United Nations University Institute for Water, Environment, and Health [34] for the climate change aspect. For the exposure indicator, population density, land cover, rainfall and temperature were used, as shown in Table 1.

First, the exposure component was reclassified to represent values ranging from 0 to 1. Twelve monthly temperature and precipitation layers were reclassified to determine exposure (assigned a value of 1) and no exposure (assigned a value of 0) for the three scenarios.

Second, the susceptibility component layers were normalized in order to obtain values ranging from 0 to 1, using the minimum/maximum approach used in a previous study in Malaysia [24] as shown below:

(1) $$\mathrm{Susceptibility}\;{\mathrm{component}}_{x}\;=\;\left(x-x_{\min}\right)/\left(x_{\max}-x_{\min}\right)$$

where x = the indicator while max and min represent the largest and smallest observed values, respectively.

The SMCE procedure included the following: the selection of the problem, generation of factor maps, followed by standardization using the goal and benefit options in the SMCE menu in ILWIS. After this, weights were applied to the factor maps. A weighting of 75% was applied to the exposure component, while 12.5% was applied to susceptibility and adaptive capacity indicators, respectively, as used in a previous study in Malaysia [24]. The maps generated had a range of values from 0 to 1, with values closer to 1 (red) representing very high vulnerability and values closer to 0 (green), indicating very low vulnerability. Specifically, the classification was conducted based on the following: 0–0.2 (very low vulnerability), 0.2–0.4 (low vulnerability), 0.4–0.6(moderate vulnerability), 0.6–0.8 (high vulnerability), and 0.8–1.0 (very high vulnerability).

## 3. Results

**Figure 2:**
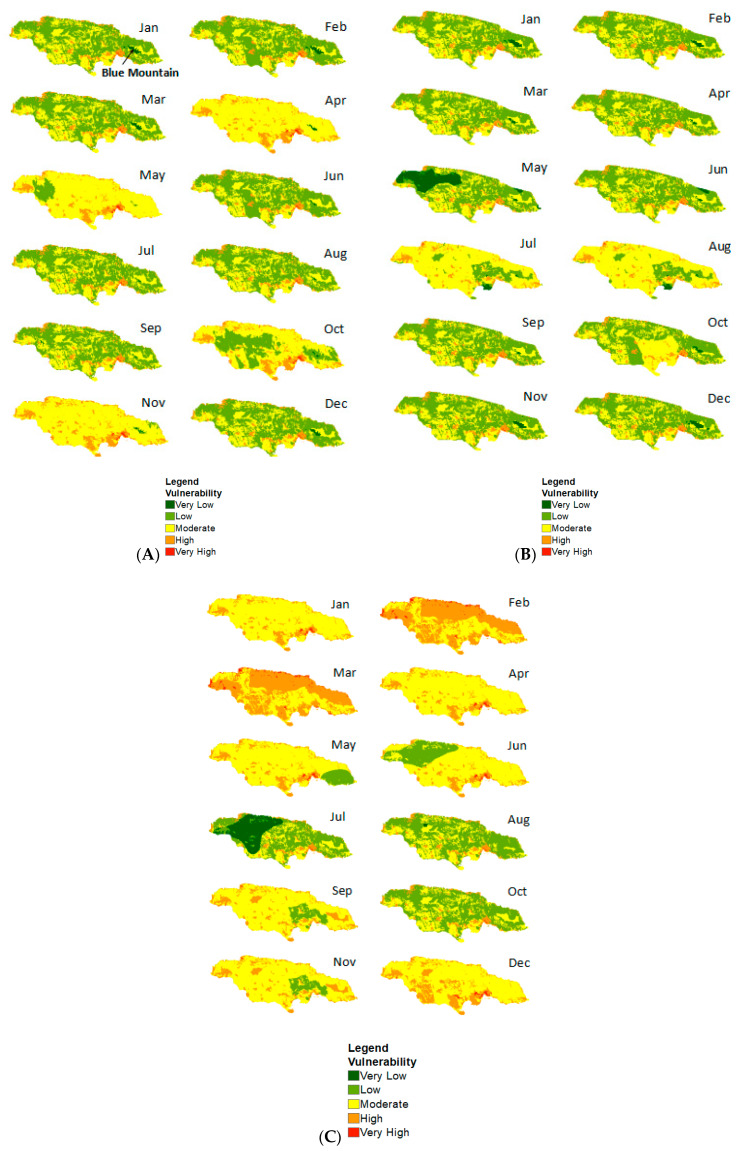
Results from the dengue vulnerability spatial multi-criteria evaluation (SMCE) scenarios: (**A**) WorldClim rainfall and temperature data 1970–2000; (**B**) Climate Hazard Group InfraRed Precipitation with Station data (CHIRPS) rainfall and Moderate Resolution Imaging Spectroradiometer (MODIS) land surface temperature (LST) for 2002 to 2016; (**C**) Representative Concentration Pathway (RCP) 8.5 climate change projection for 2030.

The results from the first scenario with the WorldClim rainfall and temperature dataset for 1970–2000 are shown in Figure 2A. As indicated, the only location with very low vulnerability (dark green) to dengue seems to be in the Blue Mountain, which has an elevation of about 2256 m and is the highest point in Jamaica. However, the extent with lower vulnerability varies monthly, with a significant reduction from May until October, and a larger extent from November to April. Figure 2A also shows that rural areas generally have a low vulnerability (light green), although this alters monthly. For example, the vulnerability in rural areas becomes moderate in April, May, and November. For the month of October, low vulnerability is mostly concentrated along eastern, central, and western sections of the island. Likewise, a low level of vulnerability is experienced in May, especially in the west. Further, areas with higher vulnerability (red) are situated in urban areas or regions with inhabitants.

In the scenario with CHIRPS and LST (Figure 2B), vulnerability to dengue varies monthly and spatially. Similar to Scenario A, lower vulnerability is mostly visible in the Blue Mountain, although the extent is reduced from July to September. January to May and November to December seem to be the months with very low vulnerability in the Blue Mountain. However, a larger area with low vulnerability in the west takes place in May. Likewise, July and August are linked to lower vulnerability in Southern Clarendon. In contrast, very high to high vulnerability is illustrated in urban areas, especially from January to June and September to December. It is also evident that moderate vulnerability increases from July and August, especially in rural areas.

As it relates to the RCP 8.5 Climate Change Scenario for 2030 (Figure 2C), similar levels of vulnerability are shown for January to April and December, ranging from high to very high.

Nonetheless, vulnerability for February and March follows a similar pattern, with high vulnerability along the north coast and moderate vulnerability east to west. Moreover, vulnerability is similar for September and November, with low levels of vulnerability in Saint Catherine and moderate to high vulnerability elsewhere.

**Figure 3:**
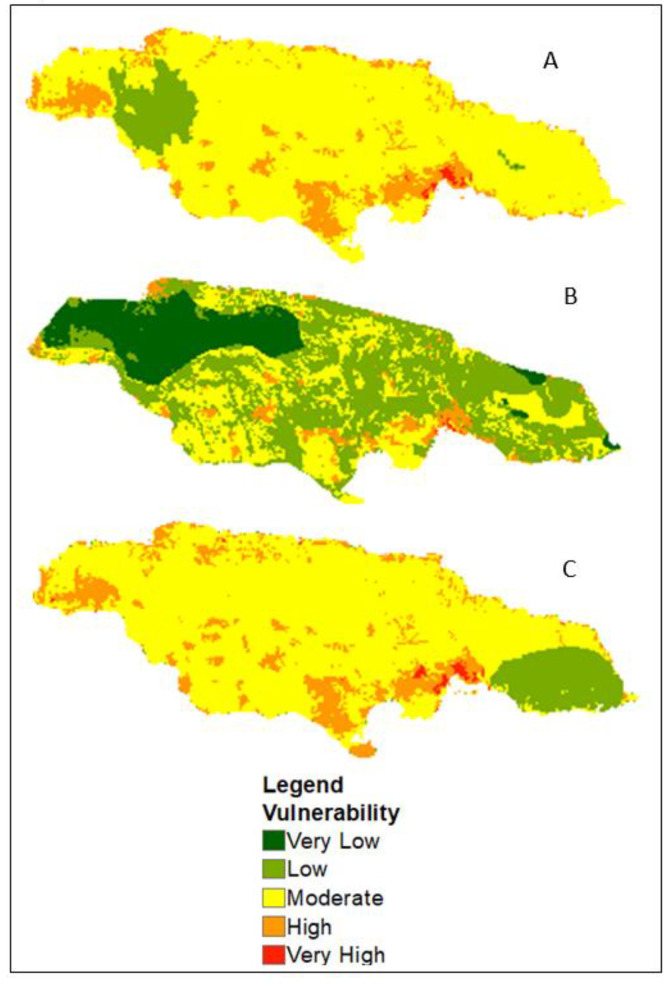
Dengue vulnerability with (**A**) WorldClim dataset, (**B**) CHIRPS rainfall and LST, and (**C**) climate change projection for 2030 for the month of May.

**Figure 4:**
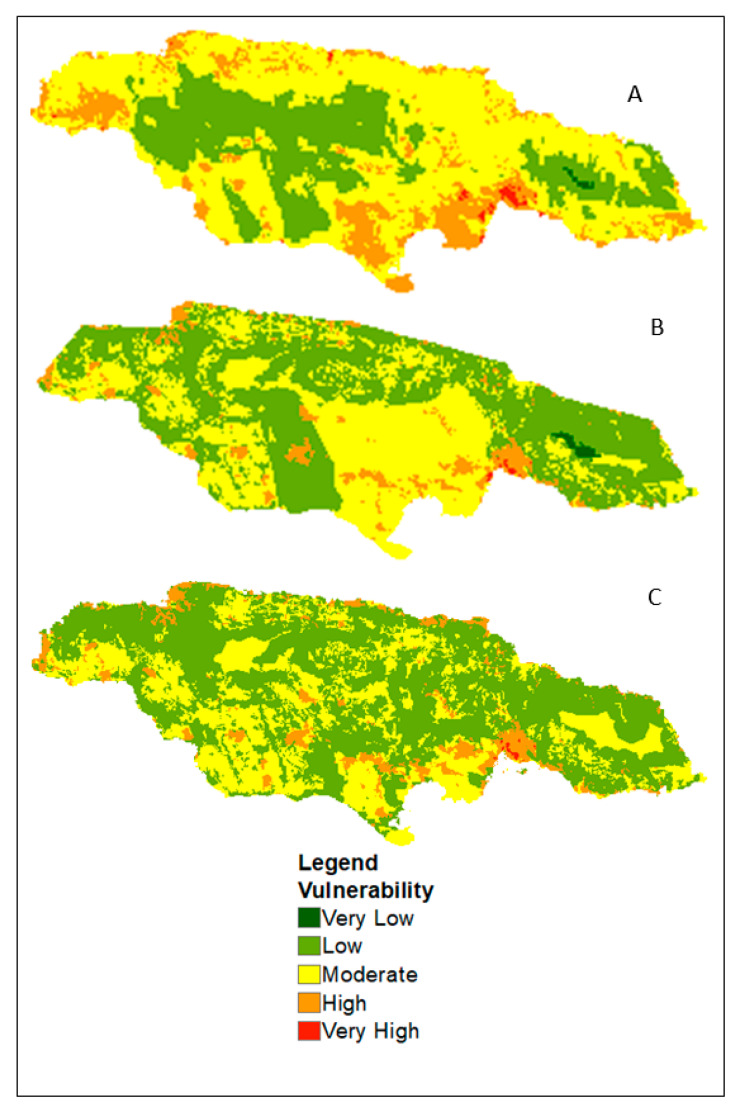
Dengue vulnerability with (**A**) WorldClim dataset (**B**) CHIRPS rainfall and LST, and (**C**) and climate change projection for 2030 for the month of October.

In order to get a better understanding of the variations among the three scenarios, comparisons were performed for the months of May (Figure 3) and October (Figure 4). For May, both the A and B scenarios (the WorldClim, CHIRPS, and LST) show a slightly similar trend for vulnerability to dengue in the west, and in Blue Mountain. While low vulnerability is illustrated in the west for Scenario A, low vulnerability will most likely occur in the eastern section of Jamaica under climate change as indicated in Scenario C. Also, only Scenario B demonstrated a larger extent for low to very low vulnerability. Furthermore, the low to very low vulnerability in the Blue Mountain in Scenarios A and B is non-existent under climate change conditions (Scenario C). Moreover, the spatial extent for Scenarios A and C follows a similar trajectory in comparison to Scenario B.

The scenarios for the month of October (Figure 4) show more areas with low vulnerability to dengue in Scenario A in the central, eastern and western end of the island. In both Scenarios A and B, low to very low vulnerability is experienced in the Blue Mountain which is non existent in the scenario under climate change (C). This implies a change in the range of dengue transmission at high altitude in October.

## 4. Discussion

One of the hypotheses in this research is that urban areas would have higher vulnerability to dengue than rural areas. In agreement with our hypothesis, studies in Malaysia [24], Vietnam [35], and Dominica in the Caribbean [36], which also utilized the WADI framework, reported similar results. The predominance of dengue in urban areas in Jamaica could be due to the socio-economic and environmental conditions and public health policies that promote the presence of the dengue vector. The haphazard manner in which planning is done in Jamaica results in inequality between urban and rural areas, thus creates under-development in the latter. This therefore leads to migration into urban areas. Currently, about 54% of Jamaicans live in urban areas, which are expected to increase to 58% by 2030 [37].

As urbanization increases, pressure is placed on health services in middle-income countries because of inadequate resources [38]. This inadequacy to meet the needs of the urban populace has been described in terms of “maldevelopment” [39]. Specifically, poor people in urban areas are known to be at great risk. According to the United Nations Economic and Social Commission for Asia and the Pacific (ESCAP) and UNISDR reports, urban poverty in developing countries normally leads to deaths, property loss, and displacement of people [40]. Furthermore, the insufficient resources among the poor are normally acquired through accumulation by dispossession, a term coined by David Harvey, which makes them unable to mitigate diseases effectively [41].

Moderate vulnerability to dengue in some rural areas could result from people occupying marginal lands due to the unavailability of housing. In Jamaica, squatting is a major problem due to urbanization and inadequate housing solutions. According to the 2008 Rapid Assessment of Squatting Report of Jamaica, only 37% of the respondents in squatter communities had access to garbage collection services and a significant number of squatters still dump their garbage [42]. Consequently, the improper disposal of garbage can lead to the creation of mosquito habitat. Research conducted in Jamaica [20] indicated that tins, tyres, and plastic containers were some of the main breeding sites for the *Aedes aegypti* mosquito in Jamaica.

In general, it has been ascertained that socio-economic variables such as population growth, movement of people, ineffective public health policy, access to health care, rapid urbanization, globalization, and inadequate domestic water supply lead to dengue infection [43,44,45]. Studies have shown how temperature and urbanization create urban heat islands that promote the spread of dengue [46]. Furthermore, dengue hotspots have been located among individuals working as domestic workers, living in deplorable housing, using containers to store water, and proximity to improper waste disposal [47]. In Jamaica, the improper storage of water in containers during drought has been identified as one of the breeding sites for the *Aedes aegypti* mosquito [21].

High vulnerability to dengue in urban areas could also result from the climatic conditions that favor the presence of the dengue vector. Climatic factors such as humidity and vapor pressure [48], though not utilized in this study, can influence dengue occurrence. In many studies, precipitation and temperature have been linked to dengue [49]. While temperature ranging from 20 to 30 °C is suitable for the presence of the *Aedes aegypti* mosquito in some countries [50], elsewhere, a minimum temperature of 11.9 °C has been found to be ideal in other locations [51]. Moreover, rainfall creates mosquito habitat and is one of the precursors for vector germination, but heavy precipitation can destroy breeding sites [52]. The foregoing shows how dengue can be easily transmitted in Jamaica based on the ideal temperature and rainfall that create mosquito habitat. Additionally, urban areas tend to be hotter than rural areas, hence the propensity for high vulnerability in the former.

The second hypothesis suggests that the geographical range for transmission of dengue would likely change under climate change conditions. This research highlighted the possibility of expansion of dengue in high elevation, which has been observed in other studies. In Nepal, two studies [53,54] indicated a shift in the spread of dengue fever in higher altitudes. Even though some research has outlined the possibility for expansion of the dengue in new areas as a result of climate change [55,56,57], there has been a reduction in habitat suitability in others due to the extreme conditions [58]. However, the *Aedes aegypti* mosquito has been adapting to a changing climate, as the vector, in one instance, changed breeding sites from drums to septic tanks in order to adapt to high temperatures [59].

One of the limitations of this research is the lack of access to time series dengue data to validate the results. However, since the goal of this investigation is to show the usefulness of publicly available spatial data in the study of dengue in countries like Jamaica where dengue data is sparse, and not necessarily to produce a validated index, the results presented can be useful for vector control as it shows how the vector responds under different conditions. The second limitation in the vulnerability assessment is the application of weights since the decision-makers may apply their preference for particular variables, resulting in bias [60].

Another disadvantage is the quality and scale of the data used. The results indicate that depending on the data source used, dengue vulnerability can vary, which can have implications for vector control. Scenarios A and B are based on past and current climate conditions, and, as shown in the comparison for May and October, the level of vulnerability differed slightly. Likewise, the spatial extent for Scenarios A and C are more alike than Scenario B. Also, the use of the climate change scenario showed how dengue vulnerability might increase in high altitudes. Since climate change is a prognosis, there is still some level of uncertainty about its effect on the spread of dengue; however, its incorporation in the analysis can provide insight as it relates to the future occurrence of the disease.

It is evident that the different scales used might have an impact on the overall result; however, it has been established that the temporal and spatial resolutions of data used in vulnerability assessments are not always the same [61]. Therefore, data are often aggregated to a particular level or used based on availability. Besides, the WADI framework was specifically designed to make use of this limitation. One of the advantages of the WADI framework is the ability to integrate data at different spatio-temporal scales [62].

## 5. Conclusions

In this study, the WADI Framework was used to assess vulnerability to dengue in Jamaica under past, current, and future climate change conditions using three different scenarios. The results showed high to very high vulnerability for urban areas and places where people reside in all the scenarios presented. A very low level of vulnerability was depicted in the Blue Mountains in most of the scenarios under current conditions; however, this level of vulnerability might be altered under climate change based on the RCP8.5 scenario for 2030. This implies a change in the climatic factors that will promote the spread of dengue at high altitudes.

As it relates temporally, April, May, and November are associated with moderate vulnerability in rural areas for the first scenario with the WorldClim dataset. However, high to very high vulnerability was illustrated in urban areas, especially from January to June and September to December, for the second scenario with the CHIRPS and LST data. It was also evident that moderate vulnerability increases between July and August, especially in rural areas. For the third scenario with climate change based on RCP 8.5 up to 2030, vulnerability for February and March followed a similar pattern with high vulnerability along the north coast and moderate vulnerability from east to west.

This research accepts the hypothesis that higher vulnerability to dengue would occur in urban areas compared to rural areas. In this study, urban areas were shown to be more favorable for dengue transmission due to the socio-economic and climate conditions that are present in these locations. Likewise, the other hypothesis that climate change would likely alter the geographical range of dengue on the island is accepted. The results generated from this research show the possibility of expansion of dengue in high elevations under climate change. However, climate change can also create less favorable conditions for the survival of the *Aedes aegypti* mosquito in some areas due to the harsh conditions.

Nevertheless, care should be taken when interpreting the results since different resolutions and scales were utilized. Similarly, alteration of the vulnerability scale can change the outcome of the study. Nonetheless, results from this analysis can be useful for vector control strategies and the allocation of resources in areas with high to very high vulnerability to dengue. Furthermore, the results show how the mosquito vector is likely to respond based on the socio-economic and climate conditions under different scenarios.

## Figures and Tables

**Table 1:** Exposure indicator used for Water-Associated Disease Index (WADI).

Exposure	Dimension	Value	Source
Population Density (per 1000 km^2^)	<0.1	0	2011 Census, STATIN
	>0.1 to <0.15	0.25	
	>0.15 to <0.25	0.50	
	>0.25 to <0.30	0.75	
	>0.30	1.0	
Land cover component	Urban	1	Forestry Department of Jamaica
	Agricultural/plantation	0.50	
	Mixed vegetated/agricultural	0.25	
	Forest	0	
Temperature	Maximum monthly temperature	>20 and ≤34 °C: linear increase in exposure up to 1; ≤20 or >34 °C: 0 exposure	WorldClim, MODIS and CCCCC
Precipitation	Monthly cumulative precipitation	<300 mm precipitation: linear increase in exposure up to 1; >300 mm monthly precipitation: 0 exposure	WorldClim, MODIS and CCCCC

Source: Adapted from the study in Malaysia [24].

**Table 2:** Susceptibility indicator used for the WADI Index.

	Components	Dimension	Source
Individual	Age under 15	% of population under 15 years	2011 Census, STATIN
	Age over 65 years	% of population over 65 years	2011 Census, STATIN
Community	Housing quality	Number of housing living in squatter settlement per parish	2011 Census, STATIN
	Piped water	% of households using piped water per parish	2011 Census, STATIN
	Sanitation	% of household using water closet per parish	2011 Census, STATIN
	Garbage Collection	% of household using public and private garbage collection system per parish	2011 Census, STATIN
	Lack of Education	% of the population with no form of schooling	2011 Census, STATIN

Source: Modified from the study in Malaysia [24].

**Table 3:** Adaptive capacity.

	Components	Dimension	Source
Community	Health care access	% of household >5 km from health clinic per parish	Ministry of Health of Jamaica
	Female education Level	% of females completing secondary education per parish	2011 Census, STATIN

Source: Adapted from the study in Malaysia [24].
